# Clinical and Metagenomic Characterization of Neurological Infections of People With Human Immunodeficiency Virus in the Peruvian Amazon

**DOI:** 10.1093/ofid/ofad515

**Published:** 2023-10-27

**Authors:** Hannah E Steinberg, Prashanth S Ramachandran, Andrea Diestra, Lynn Pinchi, Cusi Ferradas, Daniela E Kirwan, Monica M Diaz, Michael Sciaudone, Annie Wapniarski, Kelsey C Zorn, Maritza Calderón, Lilia Cabrera, Viviana Pinedo-Cancino, Michael R Wilson, Cesar Ramal Asayag, Robert H Gilman, Natalie M Bowman, Toxoplasmosis Working Group in Peru, Catherine Apaza, Melanie Ayachi, Oliver A Bocanegra, Jeroen Bok, Linda Chanamé Pinedo, Marilly Donayre Urquizo, Renzo Gutierrez-Loli, Gaston Pinedo, Grace Trompeter, Sory Vazquez, Deanna Zhu

**Affiliations:** Department of Microbiology and Immunology, University of Illinois Chicago, Chicago, Illinois, USA; Department of Infectious Diseases, The Peter Doherty Institute for Immunity and Infection, University of Melbourne, Melbourne, Australia; Department of Neurology, The Royal Melbourne Hospital, University of Melbourne, Melbourne, Australia; Department of Neurology, St. Vincent’s Hospital, University of Melbourne, Melbourne, Australia; Weill Institute for Neurosciences, Department of Neurology, University of California, SanFrancisco, San Francisco, California, USA; Laboratorio de Investigación en Enfermedades Infecciosas, Universidad Peruana Cayetano Heredia, Lima, Peru; Asociación Benéfica Prisma, Lima, Peru; Emerging Diseases and Climate Change Research Unit, School of Public Health and Administration, Universidad Peruana Cayetano Heredia, Lima, Peru; Department of Medicine and Epidemiology, School of Veterinary Medicine, University of California, Davis, Davis, California, USA; Institute for Infection and Immunity, St George's, University of London, London, United Kingdom; Department of Neurology, School of Medicine, University of North Carolina at Chapel Hill, Chapel Hill, North Carolina, USA; Division of Infectious Diseases, Tulane University School of Medicine, New Orleans, Louisiana, USA; Weill Institute for Neurosciences, Department of Neurology, University of California, SanFrancisco, San Francisco, California, USA; Weill Institute for Neurosciences, Department of Neurology, University of California, SanFrancisco, San Francisco, California, USA; Laboratorio de Investigación en Enfermedades Infecciosas, Universidad Peruana Cayetano Heredia, Lima, Peru; Asociación Benéfica Prisma, Lima, Peru; Laboratorio de Investigación de Productos Naturales Antiparasitarios de la Amazonía, Centro de Investigación de Recursos Naturales, Universidad Nacional de la Amazonía Peruana, Iquitos, Peru; Faculty of Human Medicine, Universidad Nacional de la Amazonía Peruana, Iquitos, Peru; Weill Institute for Neurosciences, Department of Neurology, University of California, SanFrancisco, San Francisco, California, USA; Department of Clinical Sciences, Universidad Nacional de la Amazonía Peruana, Iquitos, Peru; Department of Infectious and Tropical Diseases, Hospital Regional de Loreto, Iquitos, Peru; Department of International Health, Bloomberg School of Public Health, Johns Hopkins University, Baltimore, Maryland, USA; Division of Infectious Diseases, School of Medicine, University of North Carolina at Chapel Hill, Chapel Hill, North Carolina, USA

## Abstract

**Background:**

Neurological opportunistic infections cause significant morbidity and mortality in people with human immunodeficiency virus (HIV) but are difficult to diagnose.

**Methods:**

One hundred forty people with HIV with acute neurological symptoms from Iquitos, Peru, were evaluated for cerebral toxoplasmosis with quantitative polymerase chain reaction (qPCR) of cerebrospinal fluid (CSF) and for cryptococcal meningitis with cryptococcal antigen test (CrAg) in serum or CSF. Differences between groups were assessed with standard statistical methods. A subset of samples was evaluated by metagenomic next-generation sequencing (mNGS) of CSF to compare standard diagnostics and identify additional diagnoses.

**Results:**

Twenty-seven participants were diagnosed with cerebral toxoplasmosis by qPCR and 13 with cryptococcal meningitis by CrAg. Compared to participants without cerebral toxoplasmosis, abnormal Glasgow Coma Scale score (*P* = .05), unilateral focal motor signs (*P* = .01), positive Babinski reflex (*P* = .01), and multiple lesions on head computed tomography (CT) (*P* = .002) were associated with cerebral toxoplasmosis. Photophobia (*P* = .03) and absence of lesions on head CT (*P* = .02) were associated with cryptococcal meningitis. mNGS of 42 samples identified 8 cases of cerebral toxoplasmosis, 7 cases of cryptococcal meningitis, 5 possible cases of tuberculous meningitis, and incidental detections of hepatitis B virus (n = 1) and pegivirus (n = 1). mNGS had a positive percentage agreement of 71% and a negative percentage agreement of 91% with qPCR for *T gondii*. mNGS had a sensitivity of 78% and specificity of 100% for *Cryptococcus* diagnosis.

**Conclusions:**

An infection was diagnosed by any method in only 34% of participants, demonstrating the challenges of diagnosing neurological opportunistic infections in this population and highlighting the need for broader, more sensitive diagnostic tests for central nervous system infections.

## Teaser

Neurological opportunistic infections in HIV-infected patients are difficult to diagnose. We describe clinical features of *Toxoplasma* encephalitis and cryptococcal meningitis in Peruvians with HIV and compared diagnostic performance of metagenomic next-generation sequencing with that of *Toxoplasma* qPCR and cryptococcal lateral flow assay.

Approximately 60% of people with human immunodeficiency virus (PWH) develop a neurological complication during their lifetime [1]. Etiologies include opportunistic infections (OIs), other infections, malignancy, medication toxicity, and complications of human immunodeficiency virus (HIV) itself such as dementia, myelopathy, and sensorimotor neuropathy. While antiretrovirals (ARVs) diminish the risk of OIs and HIV-associated neurologic complications, undiagnosed and untreated PWH remain at risk.

Neurological OIs have decreased in incidence since implementation of ARVs; however, they remain significant sources of morbidity and mortality [1]. Cerebral toxoplasmosis and cryptococcal meningitis are 2 of the most common neurological OIs in PWH worldwide and in Peru, where an autopsy study found that *Toxoplasma* and *Cryptococcus* were the second and third most common neurological pathogens in PWH [2, 3]. Cerebral toxoplasmosis is caused by reactivation of latent *Toxoplasma gondii* infection in the brain. Definitive diagnosis is by brain biopsy, which is rarely performed because of associated risks and need for neurosurgical services. Thus, most persons are diagnosed in a clinically accepted manner, including clinicians treating empirically and pursuing other diagnoses if there is poor response to treatment after 2 weeks [4–6]. The standard-of-care laboratory diagnosis, quantitative polymerase chain reaction (qPCR) of cerebrospinal fluid (CSF), while highly specific, lacks sensitivity (35%–70%) [4–6]. Meningitis caused by *Cryptococcus neoformans* is easier to diagnose: A commercially available lateral flow assay (LFA) has 99% sensitivity and specificity for cryptococcal antigen in serum and CSF as compared to cryptococcal latex agglutination [7, 8].

Neurological conditions in PWH often have nonspecific signs and symptoms like headache, confusion, fever, focal deficits, and mental status changes, making diagnosis difficult without advanced diagnostic testing. Advances in metagenomic next-generation sequencing (mNGS) have recently begun aiding clinicians in complex diagnostic scenarios [9].

This study describes clinical and laboratory findings of persons with cerebral toxoplasmosis and cryptococcal meningitis and explores mNGS to diagnose these diseases and other neurological infections. Improved understanding of the range of diagnoses and their associated clinical findings in this patient group will aid both clinicians and researchers.

## METHODS

### Patient Consent Statement

Written informed consent was obtained from each patient or their healthcare proxy. The design of the work has been approved by local ethical committees of the Hospital Regional de Loreto, Iquitos, Peru (ID-044-CIEI-2019) and Universidad Peruana Cayetano Heredia, Lima, Peru (100804), and by the institutional review boards of the international supporting sites (University of North Carolina at Chapel Hill [#14-1253], Johns Hopkins University [9528], and University of California, San Francisco [IRB 13-12236]).

### Study Site and Population

Participants were recruited between August 2014 and March 2020 from Hospital Regional de Loreto in the city of Iquitos, Peru (population ∼400 000) as part of a multisite cohort of PWH designed to study neurological OIs and develop diagnostic tests. Hospital Regional is a public hospital that serves the department of Loreto (population >800 000). Loreto's adjusted HIV mortality rate (21.2/100 000) is the highest in Peru (national average 3.9/100 000) [10].

Inclusion criteria were age ≥18 years, laboratory-confirmed HIV infection, onset of neurological symptoms within the prior 6 months, and written informed consent. Participants completed a questionnaire about demographic and medical history and underwent physical examination, including neurological examination. Blood and urine were obtained, and remnant CSF was collected if lumbar puncture was performed by the participant's medical providers. Hospital course and laboratory values were abstracted from the medical record, and diagnostic testing was performed as described below and in the Supplementary Methods.

### Definition of Cases

Cerebral toxoplasmosis cases were defined for the purposes of this study by positive *T gondii* qPCR (cycle threshold [Ct] of ≤37; see Supplementary Methods), and cryptococcal meningitis cases were defined by positive cryptococcal antigen (CrAg) LFA (IMMY, Norman, Oklahoma) in either serum or CSF as described in the Supplementary Methods.

### CSF Metagenomic Next-Generation Sequencing

Details of mNGS library preparation and sequencing are described in the Supplementary Methods. In brief, pathogen RNA and DNA were extracted and sequencing libraries prepared using commercial kits. Sequencing was performed using New England Biolabs’ (Ipswich, Massachusetts) NEBNext Ultra II RNA and DNA library preparation kits as previously described, and sequencing libraries were sequenced on Illumina NovaSeq [11]. The open-source, cloud-based metagenomics pipeline CZ ID was used for mNGS analysis [12]. Positive samples by mNGS were defined as 1 or more sequences aligning to a pathogen, with the entire sequence aligning (with no alignment to any other species) to the pathogen genome with ≥99% nucleotide sequence identity, which was confirmed through Basic Local Alignment Search Tool (BLAST) [11]. Several pathogens can be incidentally detected on CSF mNGS without clear disease association; these include Epstein-Barr virus, cytomegalovirus, human herpesvirus 6, and human herpesvirus 7. In these cases, only clear outlier cases defined as log_10_ (reads Per Million [rPM]) 2-fold greater than the mean abundance of the entire cohort were presumed pathogenic [11].

### Computed Tomography

A neurologist reviewed all head CT scans and recorded data in a standardized questionnaire (Supplementary Table 1). History, physical examination findings, CD4 and CD8 cell counts, and HIV viral load (VL) were provided, but microbiological results were not.

### Statistical Analysis

Univariate relationships between each outcome (cerebral toxoplasmosis, cryptococcal meningitis, and mortality) and demographic, clinical, laboratory, and radiographic covariates were explored using χ^2^, Fisher exact, or Kruskal-Wallis tests for categorical variables and Mann-Whitney *U* test for numerical variables. Poisson regression with robust variance to estimate prevalence ratios (PR). Poisson regression was selected because of the ratio of positive to negative participants for the outcome variables. Data were analyzed using Stata version 15.0 software (StataCorp LLC, College Station, Texas).

## RESULTS

**Figure 1.**
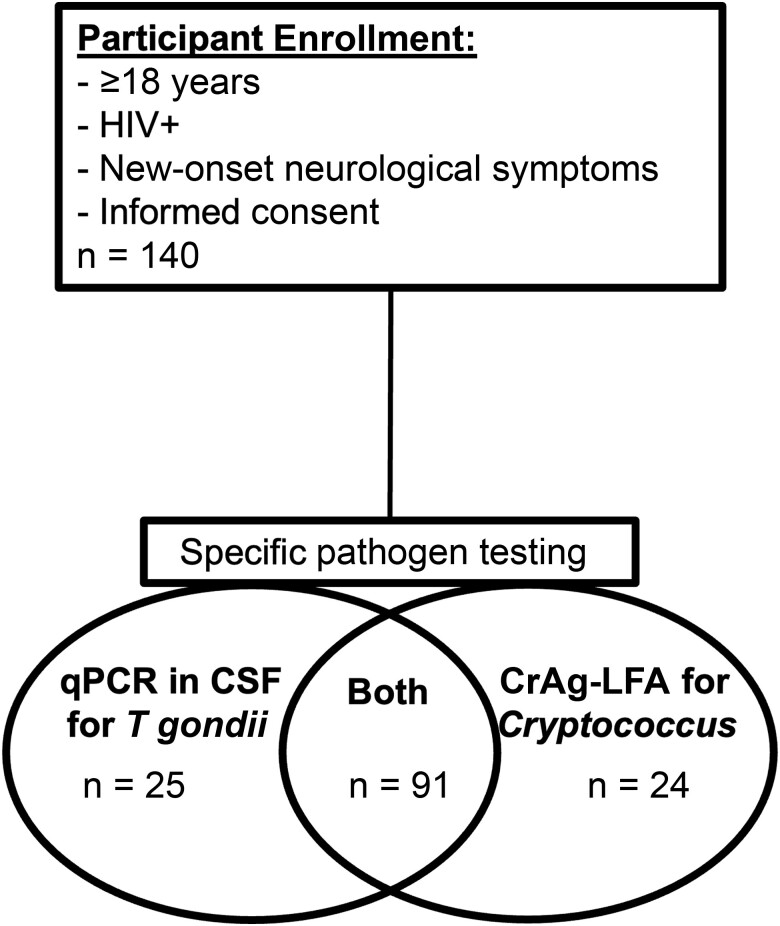
Study participant enrollment and testing. Of 140 participants, 91 were tested for both *Toxoplasma gondii* and *Cryptococcus neoformans,* 25 received only *T gondii* quantitative polymerase chain reaction (qPCR) testing, and 24 received only cryptococcal antigen (CrAg) testing. Participants received incomplete testing due to supply chain problems with the CrAg lateral flow assay or lack of cerebrospinal fluid specimen for *T gondii* qPCR. Abbreviations: CrAg-LFA, cryptococcal antigen lateral flow assay; CSF, cerebrospinal fluid; HIV+, human immunodeficiency virus positive; qPCR, quantitative polymerase chain reaction.

One hundred forty PWH with specific pathogen testing were included in the analyses (Figure 1, Table 1). Median age was 34 years (interquartile range [IQR], 28–42 years). Median time since HIV diagnosis was 1.3 years (IQR, 10 days–6.15 years), and 40% of participants were diagnosed with HIV ≤90 days prior to enrollment. Fifty-two percent of participants were taking ARVs. CD4 cell count and HIV VL within 1 year of study participation were only available for 54% and 49% of participants, respectively: median CD4 count was 81 (IQR, 34–216) cells/mL, and median VL was 58 500 (IQR, 354–300 500) copies/mL. Twenty participants (14%) had undetectable HIV VL. Ninety-seven percent of participants had positive *T gondii* immunoglobulin G serology (Table 1). Sixty-nine percent of participants (n = 96) had a head CT, though only 15 received contrast.

**Table 1.** Baseline Demographic, Human Immunodeficiency Virus, and Laboratory Findings for Included Patients

Characteristic	no./No. (%) or Median (IQR)
Demographics	
Age, y	34 (28–42)
Sex, male	107/138 (78%)
Years of education	9 (7–11)
Location (urban)	78/136 (57%)
HIV	
Time since diagnosis	1.3 y (10 d–6.15 y)
<3 mo	53/133 (40%)
3–6 mo	5/133 (4%)
6 mo–1 y	7/133 (5%)
1–2 y	17/133 (13%)
3–4 y	13/133 (10%)
5–9 y	26/133 (20%)
≥10 y	12/133 (9%)
Treatment (% yes)	71/138 (52%)
FTC ± TDF ± EFV	15/71 (21%)
EFV ± 3TC ± TDF	12/71 (17%)
ABC ± 3TC ± EFV	8/71 (11%)
3TC ± ZDV ± EFV	8/71 (11%)
Other	11/71 (15%)
Unknown	17/71 (24%)
Frequency of forgetting treatment	
Once per week	6/53 (11%)
Twice per week	6/53 (11%)
3 times per week	3/53 (6%)
4 times per week	18/53 (34%)
Unknown/missing	20/53 (38%)
Standard blood laboratory findings	
Leukocytes/mm^3^	5400 (3570–7770)
Lymphocytes/mm^3^	820 (485–1525)
Neutrophils/mm^3^	3690 (2300–4900)
Eosinophils/mm^3^	40 (10–140)
Platelets/mm^3^	266 000 (190 000–339 000)
Hematocrit(%)	31 (27–35)
CD4 cells/mm^3^	81 (34–216)
CD8 cells/mm^3^	833 (472–1113)
Log Viral Load/mm^3^	58 500 (354–300 500)
Diagnostic laboratory findings	
Toxoplasmosis serology (% positive)	128/132 (97%)
*Toxoplasma gondii* qPCR CSF (% positive)	27/115 (24%)
CrAg-LFA serum (% positive)	8/92 (9%)
CrAg-LFA CSF (% positive)	7/56 (13%)
Imaging findings	
Neurological toxoplasmosis	
Probable	16/98 (16%)
Possible	5/98 (5%)
Less likely	6/98 (6%)
Meningitic process	
Probable	11/98 (11%)
Possible	9/98 (9%)
Less likely	6/98 (6%)
Survival (% living)	53/114 (46%)

Abbreviations: 3TC, lamivudine; ABC, abacavir; CSF, cerebrospinal fluid; CrAg-LFA, cryptococcal antigen lateral flow assay; EFV, efavirenz; FTC, emtricitabine; HIV, human immunodeficiency virus; IQR, interquartile range; qPCR, quantitative polymerase chain reaction; TDF, tenofovir disoproxil fumarate; ZDV, zidovudine.

Of 115 participants (82%) tested for cerebral toxoplasmosis by qPCR, 27 (23%) were positive (Table 2). Mortality was 40% in those with cerebral toxoplasmosis and 45% in qPCR-negative participants (PR, 1.22 [95% confidence interval {CI}, .61–2.44]). There were no significant differences in demographic characteristics between positive and negative participants. The qPCR-positive participants were more likely to have an abnormal Glasgow Coma Scale (GCS) score (<15), (*P* = .05), focal motor signs (*P* = .01), or positive Babinski reflex (*P* = .01) than negative participants. All *T gondii* qPCR-positive patients reported missing their ARVs at least once per week (*P* = .04). There was a nonsignificant trend toward lower median CD4 count in qPCR-positive (52 [IQR, 21–81] cells/mm^3^) versus qPCR-negative participants (141 [IQR, 34–325] cells/mm^3^) (*P* = .06). Median CD8 T-cell counts were significantly higher in qPCR-positive patients (1151 [IQR, 676–1467] cells/mL) than qPCR-negative patients (776 [IQR, 473–999] cells/mL) (*P* = .03).

**Table 2.** Demographic and Clinical Factors of *Toxoplasma gondii* Quantitative Polymerase Chain Reaction in Cerebrospinal Fluid–Positive and –Negative Patients

	*Toxoplasma*		
	Positive (n = 27)	Negative (n = 88)	
Characteristic	no./No. (%) or Median (IQR)	no./No. (%) or Median (IQR)	*P* Value^a^
Demographics			
Age, y	34 (28–40)	34 (31–42)	.59
Sex (% male)	20/26 (77%)	69/87 (79%)	.79
Years of education	11 (8–12)	9 (7–12)	.17
Location (% rural)	11/25 (44%)	39/87 (45%)	.94
Animals (% no)	8/24 (33%)	20/86 (24%)	.32
Symptoms			
Weight loss (% yes)	22/26 (85%)	79/87 (91%)	.37
Fever and chills (% yes)	21/26 (81%)	69/86 (80%)	.95
Night sweats (% yes)	16/25 (64%)	50/82 (61%)	.79
Seizures (% yes)	7/23 (30%)	24/81 (30%)	.94
Headache (% yes)	21/25 (84%)	77/86 (90%)	.45
Neck stiffness (% yes)	9/24 (38%)	17/75 (23%)	.15
Photophobia (% yes)	7/25 (28%)	27/84 (32%)	.70
Neurological exam findings			
GCS score	13.5 (9–15)	14 (14–15)	.**01**
GCS score binary (% <15)	19/26 (73%)	44/86 (51%)	.**05**
MMSE (total score)^b^	11 (9–27)	19.5 (9–26.5)	.30
IHIVDS^c^	9 (4–9)	9 (4–9)	.91
Gait abnormalities (% yes)	3/5 (60%)	10/32 (31%)	.21
Romberg sign (% positive)	3/5 (60%)	10/32 (31%)	.74
Alternating movement test (% abnormal)	3/12 (25%)	9/51 (18%)	.56
Nose to finger (% abnormal)	3/12 (25%)	12/55 (22%)	.81
Diminished global strength (% positive)	15/19 (79%)	33/68 (49%)	.**02**
Focal sensory signs (unilateral)	10/25 (40%)	20/82 (24%)	.13
Focal motor signs (unilateral)	17/25 (68%)	30/82 (37%)	.**01**
Babinski reflex (% positive)	17/26 (65%)	31/82 (38%)	.**01**
Trömner sign (% positive)	4/26 (15%)	12/82 (15%)	.93
HIV			
Time since diagnosis	2.8 y (17 d–5.8 y)	352 d (5 d–6.5 y)	.74
Treatment (% yes)	12/24 (50%)	44/84 (52%)	.84
Forget treatment (% yes)	8/8 (100%)	24/39 (62%)	.**04**
Previous opportunistic infection	8/21 (38%)	22/69 (31%)	.60
Laboratory findings (blood)			
Leukocytes/mm^3^	4760 (3200–10 000)	5710 (3755–7780)	.41
Lymphocytes/mm^3^	567 (410–880)	880 (490–1690)	.11
Hematocrit(%)	32.2 (28–34)	31.9 (27–36)	.87
CD4 cells/mm^3d,e^	52 (21–81)	141 (34–325)	.06
CD8 cells/mm^3e^	115 (68–147)	78 (47–100)	.**03**
Ratio CD4/CD8	0.09 (0.03–0.3)	0.06 (0.16–0.34)	.28
Log Viral Load/mm^3^	10.1 (8–12)	10.4 (5.8–12.5)	1.00
Toxoplasmosis serology (% positive)	26/26 (100%)	80/82 (98%)	1.00
Clinical outcomes			
Treatment with TMP-SMX (% yes)	24/27 (88%)	75/88 (85%)	.63
Survival at 6 mo (% living)	10/25 (40%)	33/71 (46%)	.58

Significant Values in bold. Abbreviations: GCS, Glasgow Coma Scale; HIV, human immunodeficiency virus; IHIVDS, International HIV Dementia Scale; IQR, interquartile range; MMSE, Mini-Mental State Examination; TMP-SMX, trimethoprim-sulfamethoxazole.

^a^ *P* values were calculated with χ^2^ tests or Fisher exact test for categorical variables and Mann-Whitney *U* test for numerical variables.

^b^Normal MMSE >25.

^c^Normal IHIVDS >10.

^d^Only individuals with a CD4 count in the last year were included in the analysis.

^e^Regression analysis completed using CD4 cells/mm^3^/100 cells/mm^3^ and CD8 cells/mm^3^/100 cells/mm^3^.

**Figure 2.**
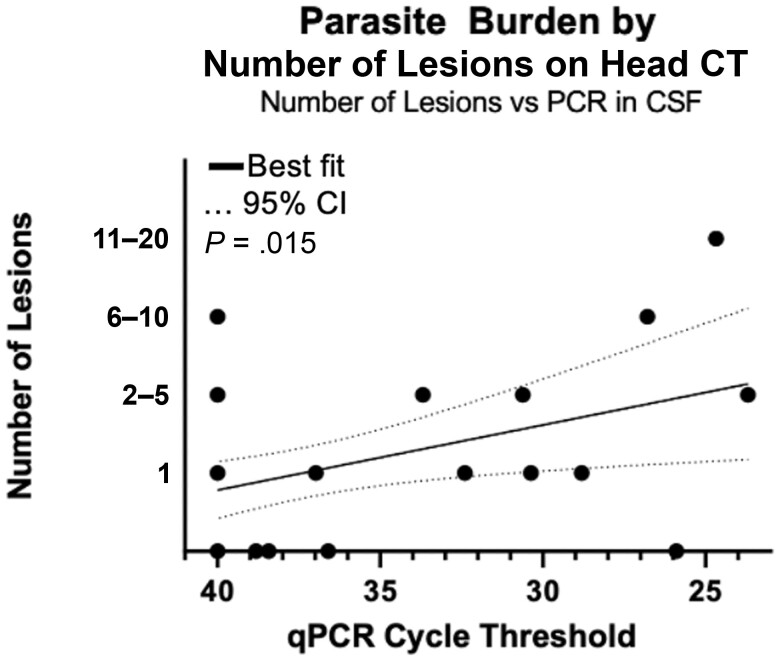
Relationship between *Toxoplasma gondii* quantitative polymerase chain reaction cycle threshold and number of lesions on head computed tomography. Abbreviations: CI, confidence interval; CSF, cerebrospinal fluid; CT, computed tomography; PCR, polymerase chain reaction; qPCR, quantitative polymerase chain reaction.

Eighteen qPCR-positive and 60 qPCR-negative patients underwent head CT (Table 3). Of 18 qPCR-positive participants’ CT scans, 10 were classified as probable, 3 possible, 4 less likely, and 1 not consistent with cerebral toxoplasmosis (Supplementary Table 1). Of 60 qPCR-negative participants’ CT scans, 6 were classified as probable, 2 possible, and 2 less likely cases of cerebral toxoplasmosis (Supplementary Table 1). qPCR-positive patients were more likely to have any lesions (*P* = .002), more lesions (*P* < .001), hyperdense lesions (*P* < .001), bilateral lesions (*P* = .01), edema (*P* < .001), mass effect (*P* < .001), white matter changes (*P* = .012), or more diffuse white matter changes (*P* < .001) (Table 3). There was a significant inverse relationship (*R*^2^ = .13, *P* = .02) between qPCR Ct and number of lesions identified on head CT (Figure 2). Only 14 scans were performed with contrast; analyses were performed stratified by receipt of contrast (Supplementary Table 2). The only difference between contrast and noncontrast groups was identification of white matter changes (Supplementary Table 2). Eight participants had follow-up CTs described in Supplementary Table 3.

**Table 3.** Head Computed Tomography Results by Quantitative Polymerase Chain Reaction of *Toxoplasma gondii*

	*Toxoplasma*					
	Positive (n = 18)	Negative (n = 60)		Poisson Regression With Robust Variance		
CT Findings	no./No. (%) or Median (IQR)	no./No. (%) or Median (IQR)	*P* Value^a^	PR	(95% CI)	*P* Value
Lesions (% yes)	16/18 (89%)	29/60 (48%)	**.001**	5.87	(1.43–23.9)	**.01**
No. of lesions						
None	2 (6%)	32 (94%)	**<**.**001**	Ref.	…	
1	5 (28%)	13 (72%)		4.72	(1.01–22.2)	.**05**
2–5	6 (33%)	12 (67%)		5.67	(1.26–25.5)	.**02**
6–10	3 (50%)	3 (50%)		8.50	(1.76–41.1)	.**01**
11–20	2 (100%)	0		…	…	
No. of hyperdense lesions						
None	8 (13%)	54 (87%)	**<**.**001**	Ref.	…	
1	5 (56%)	4 (44%)		4.31	(1.79–10.4)	.**006**
2–5	4 (67%)	2 (33%)		5.17	(2.18–12.27)	.**006**
6–10	1 (100%)	0		…	…	
No. of hypodense lesions						
None	7 (17%)	35 (83%)	.12	Ref.	…	
1	2 (17%)	10 (83%)		1.00	(.24–4.24)	1.00
2–5	5 (29%)	12 (71%)		1.77	(.65–4.83)	.27
6–10	3 (50%)	3 (50%)		3.00	(1.05–8.61)	.**04**
11–20	1 (100%)	0		6.00	(3.04–11.9)	**<**.**001**
No. of affected hemispheres						
None	3 (9%)	31 (91%)	.**01**	Ref.	…	
1	6 (25%)	18 (75%)		2.83	(.78–10.3)	.11
2	9 (45%)	11 (55%)		5.10	(1.55–16.8)	.**01**
Edema						
None	3 (7%)	38 (93%)	**<**.**001**	Ref.	…	
Minor	5 (24%)	16 (76%)		3.25	(.85–12.4)	.08
Moderate	7 (58%)	5 (42%)		7.97	(2.41–26.4)	.**001**
Severe	3 (75%)	1 (25%)		10.25	(2.98–35.3)	**<**.**001**
Mass effect						
None	6 (10%)	52 (90%)	**<**.**001**	Ref.	…	
Minor	5 (46%)	6 (55%)		4.39	(1.61–12.0)	.**004**
Moderate	6 (75%)	2 (25%)		7.25	(3.06–17.2)	**<**.**001**
Severe	1 (100%)	0		…	…	
Hydrocephalus (% yes)	1/18 (6%)	5/60 (8%)	.70	0.71	(.11–4.48)	.71
White matter changes (% yes)	9/18 (50%)	12/60 (20%)	**.01**	2.71	(1.24–5.93)	**.01**
Type of white matter changes						
None	9 (16%)	48 (84%)	.**001**	Ref.	…	
Minor	3 (25%)	9 (75%)		1.58	(.50–5.03)	.44
Moderate	6 (86%)	1 (14%)		5.43	(2.76–10.7)	**<**.**001**
Generalized	0	2 (100%)		…	…	

Significant Values in bold. Abbreviations: CI, confidence interval; CT, computed tomography; IQR, interquartile range; PR, prevalence ratio.

^a^ *P* values were calculated with χ^2^ test or Fisher exact test for categorical variables and Mann-Whitney *U* test for numerical variables.

Thirteen of 116 participants (11%) who were tested for *C neoformans* were positive for CrAg in serum or CSF. Of 32 participants tested with both CSF and serum, all had concordant results (κ = 1) (Supplementary Table 4); thus, all participants tested for *C neoformans* were included in the analysis regardless of specimen(s) tested.

Participants with and without *Cryptococcus* infection had similar demographic and clinical characteristics (Table 4). All CrAg-positive patients reported weight loss and headache. *Cryptococcus*-positive patients were more likely to report photophobia (*P* = .04) and were less likely to report night sweats than negative participants (*P* = .04). The patients with cryptococcal meningitis were significantly less likely to have focal sensory deficits (*P* < .001).

**Table 4.** Demographic and Clinical Factors of Cryptococcal Antigen–Positive and –Negative Patients

	*Cryptococcus*		
	Positive (n = 13)	Negative (n = 103)	
Characteristic	no./No. (%) or Median (IQR)	no./No. (%) or Median (IQR)	*P* Value^a^
Demographics			
Age, y	36 (32–41)	35 (29–44)	.88
Sex (% male)	12/13 (92%)	79/102 (77%)	.21
Years of education	10 (8–11)	9 (7–12)	.93
Location (% urban)	8/12 (67%)	56/100 (56%)	.48
Animals (% yes)	9/12 (75%)	69/98 (70%)	.89
Symptoms			
Weight loss (% yes)	13/13 (100%)	91/101 (90%)	.60
Fever and chills (% yes)	9/13 (69%)	83/101 (82%)	.27
Night sweats (% yes)	4/13 (31%)	61/97 (63%)	.**03**
Seizures (% yes)	5/12 (42%)	24/95 (25%)	.23
Headache (% yes)	13/13 (100%)	85/100 (85%)	.21
Neck stiffness (% yes)	4/12 (33%)	17/92 (19%)	.23
Photophobia (% yes)	7/13 (54%)	25/100 (25%)	.**03**
Neurological exam findings			
GCS score	14 (11–15)	14 (13–15)	.98
GCS score binary (% <15)	7/13 (54%)	55/102 (54%)	1.00
MMSE (total score)^b^	15 (9–27.5)	20 (9–28)	.74
IHIVDS^c^	9 (8–9)	9 (4–9)	.32
Gait abnormalities (% yes)	0/4 (0%)	15/41 (37%)	.29
Romberg sign (% positive)	2/4 (50%)	10/37 (27%)	.34
Alternating movement test (% abnormal)	1/6 (17%)	14/62 (23%)	.74
Nose to finger (% abnormal)	2/7 (28%)	14/64 (22%)	.69
Diminished global strength (% positive)	5/10 (50%)	44/81 (54%)	.80
Focal sensory signs (unilateral)	0/12 (0%)	28/98 (29%)	.**03**
Focal motor signs (unilateral)	2/13 (15%)	45/97 (46%)	.**03**
Babinski reflex (% positive)	5/13 (38%)	44/99 (44%)	.68
Trömner sign (% positive)	2/13 (15%)	13/99 (13%)	.82
HIV			
Time since diagnosis, y	0.96 (0.01–6.09)	1.09 (0.02–5.25)	.85
Treatment (% yes)	7/12 (58%)	56/98 (57%)	.94
Forget treatment (% yes)	3/6 (50%)	33/47 (70%)	.32
Previous opportunistic infection (% yes)	3/11 (27%)	29/83 (35%)	.61
Laboratory findings (blood)			
Leukocytes/mm^3^	6580 (3570–7840)	5430 (3700–7880)	.65
Lymphocytes/mm^3^	580 (285–855)	810 (470–1520)	.13
Hematocrit (%)	35.2 (31.1–36)	31.2 (26.1–34)	.**03**
CD4 cells/mm^3d,e^	52 (20–140)	141 (40–280)	.18
CD8 cells/mm^3e^	68 (51–920)	87.5 (50–132)	.26
Ratio CD4/CD8	0.08 (0.04–0.13)	0.16 (0.06–0.3)	.17
Log Viral Load/mm^3^	10.8 (8.2–11.7)	10.7 (5.8–12.6)	.92
Toxoplasmosis serology (% positive)	11/11 (100%)	96/96 (100%)	
Clinical outcomes			
Treatment with AmB + FCZ (% yes)	6/13 (46%)	28/103 (27%)	.15
Survival at 6 mo (% living)	4/9 (44%)	40/87 (46%)	.93

Significant Values in bold. Abbreviations: AmB, Amphotericin B; FCZ, Fluconazole; GCS, Glasgow Coma Scale; HIV, human immunodeficiency virus; IHIVDS, International HIV Dementia Scale; IQR, interquartile range; MMSE, Mini-Mental State Examination.

^a^ *P* values were calculated with χ^2^ test or Fisher exact test for categorical variables and Mann-Whitney *U* test for numerical variables.

^b^Normal MMSE >25.

^c^Normal IHIVDS >10.

^d^Only individuals with a CD4 count in the last year were included in the analysis.

^e^Regression analysis completed using CD4 cells/mm^3^/100 cells/mm^3^ and CD8 cells/mm^3^/100 cells/mm^3^.

Eighty-two participants (70%) with CrAg results underwent CT scan, including 9 with cryptococcal meningitis, of whom 4 were classified as probable, 1 as possible, and 4 less likely cases of cryptococcal meningitis (Supplementary Table 5). Of 74 CrAg-negative participants, 7 were classified as probable, 8 possible, and 2 less likely to have cryptococcal meningitis. The presence of lesions on head CT was associated with decreased likelihood of cryptococcal infection (PR, 0.19 [95% CI, .04–.88]).

Of 114 participants (81%) with survival data available at 6 months, 53 (46%) were alive (Table 5). Participants who died had fewer years of education (*P* = .01). Survival was not associated with diagnosis with cerebral toxoplasmosis or cryptococcal meningitis. Lower CD4 count (*P* = .005), higher VL (*P* = .05), and oral thrush (*P* < .001) were associated with increased risk of death, as was lower hematocrit level (*P* < .001). Abnormal GCS score (*P* < .001), lower Mini-Mental State Examination score (*P* < .001), positive Babinski reflex (*P* = .02), and positive Trömner sign (*P* = .01) were associated with higher mortality. Data missingness was equal between groups except in sections of the neurological examination that required active participation, where participants who died had higher rates of missing data. Supplementary Table 6 compares head CTs based on survival; only increased number of lesions was associated with increased mortality (*P* = .03).

**Table 5.** Demographic and Clinical Factors of Live and Dead Patients

	Survival		
	Alive (n = 53)	Dead (n = 61)	
Characteristic	no./No. (%) or Median (IQR)	no./No. (%) or Median (IQR)	*P* Value^a^
Demographics			
Age, y	34 (29–39)	34 (28–43)	.97
Sex (% male)	40/52 (77%)	50/60 (83%)	.39
Years of education	11 (8–12)	9 (6–11)	**.01**
Location (% urban)	29/52 (56%)	34/59 (58%)	.84
Diagnosis and treatment			
qPCR in for CSF *Toxoplasma gondii* (% positive)	10/43 (23%)	15/53 (28%)	.58
Toxoplasmosis serology (% positive)	53/53 (100%)	58/59 (95%)	1.00
CrAg for *Cryptococcus* (% positive)	4/44 (9%)	5/52 (10%)	.93
Treatment with TMP-SMX (% yes)	43/51 (81%)	53/61 (86%)	.40
Treatment with AmB + FCZ (% yes)	7/53 (13%)	21/61 (34%)	**.01**
Symptoms			
Weight loss (% yes)	44/51 (86%)	57/60 (95%)	.11
Fever and chills (% yes)	38/51 (75%)	50/60 (83%)	.25
Night sweats (% yes)	25/51 (49%)	38/55 (69%)	**.04**
Seizures (% yes)	11/51 (22%)	20/55 (36%)	.09
Headache (% yes)	46/51 (90%)	50/59 (85%)	.39
Neck stiffness (% yes)	10/47 (21%)	15/52 (29%)	.39
Photophobia (% yes)	16/50 (32%)	19/59 (32%)	.98
Neurological exam findings			
GCS score	15 (14–15)	14 (11–15)	**<.001**
GCS score binary (% <15)	16/50 (32%)	43/61 (71%)	**<.001**
MMSE (total score)^b^	26 (14–29)	11.5 (9–20)	**<.001**
IHIVDS^c^	8 (3.5–9.5)	9 (4–9)	.85
Gait abnormalities (% yes)	12/29 (41%)	3/10 (30%)	.52
Romberg sign (% positive)	8/28 (29%)	1/8 (13%)	.36
Alternating movement test (% abnormal)	6/38 (16%)	5/25 (20%)	.67
Nose to finger (% abnormal)	6/38 (16%)	7/28 (25%)	.35
Diminished global strength (% positive)	24/45 (53%)	25/43 (58%)	.65
Focal sensory signs (unilateral)	13/50 (26%)	17/58 (29%)	.70
Focal motor signs (unilateral)	23/49 (47%)	27/57 (47%)	.97
Babinski reflex (% positive)	16/49 (33%)	33/59 (56%)	**.02**
Trömner sign (% positive)	2/49 (4%)	13/59 (22%)	**.01**
HIV			
Time since diagnosis, y	2.3 (0.05–6.7)	1.95 (0.04–6.6)	.78
Treatment (% yes)	30/51 (59%)	31/61 (51%)	.35
Forget treatment (% yes)	19/27 (70%)	18/23 (78%)	.53
Previous opportunistic infection (% yes)	15/41 (37%)	14/46 (30%)	.54
Laboratory findings (blood)			
Leukocytes/mm^3^	5325 (4090–7060)	4775 (2950–7535)	.43
Lymphocytes/mm^3^	895 (675–1525)	590 (410–1290)	.11
Hematocrit (%)	33.4 (31–36)	29.3 (25.2–33.1)	**<.001**
CD4 cells/mm^3^ ^d,e^	190 (50–350)	60 (30–140)	**.01**
CD8 cells/mm^3e^	87 (65–124)	75 (45–113)	.29
Ratio CD4/CD8	0.2 (0.07–0.3)	0.1 (0.04–0.3)	.63
Log Viral Load/mm^3^	9.6 (4.4–11.8)	11 (8.5–12.7)	**.05**

Significant Values in bold. Abbreviations: AmB, Amphotericin B; CrAg, cryptococcal antigen; CSF, cerebrospinal fluid; FCZ, Fluconazole; GCS, Glasgow Coma Scale; HIV, human immunodeficiency virus; IHIVDS, International HIV Dementia Scale; IQR, interquartile range; MMSE, Mini-Mental State Examination; qPCR, quantitative polymerase chain reaction; TMP-SMX, trimethoprim-sulfamethoxazole.

^a^ *P* values were calculated with χ^2^ test or Fisher exact test for categorical variables and Mann-Whitney *U* test for numerical variables.

^b^Normal MMSE >25.

^c^Normal IHIVDS >10.

^d^Only individuals with a CD4 count in the last year were included in the analysis.

^e^Regression analysis completed using CD4 cells/mm^3^/100 cells/mm^3^ and CD8 cells/mm^3^/100 cells/mm^3^.

*Toxoplasma gondii* qPCR and CrAg-LFA provided a diagnosis for only 39 (28%) participants. To evaluate diagnostic performance and investigate other potential causes of neurological infection, mNGS was performed on CSF from 42 participants with sufficient remaining volume after diagnostic testing. Average sequencing depth was 1 278 118 reads for RNA-seq and 5 954 261 reads for DNA-seq. Of these 42, 8 were diagnosed with *T gondii* by qPCR and 6 with cryptococcal meningitis by CrAg; 28 had no diagnosis. mNGS identified potential infectious etiologies in 48% (n = 22) of the tested population, including the following organisms: *T gondii* (n = 8), *C neoformans* (n = 7), and *Mycobacterium tuberculosis* (n = 5). Additionally, mNGS detected likely nonpathogenic viruses—hepatitis B virus (HBV; n = 1) and pegivirus (n = 1).

**Figure 3.**
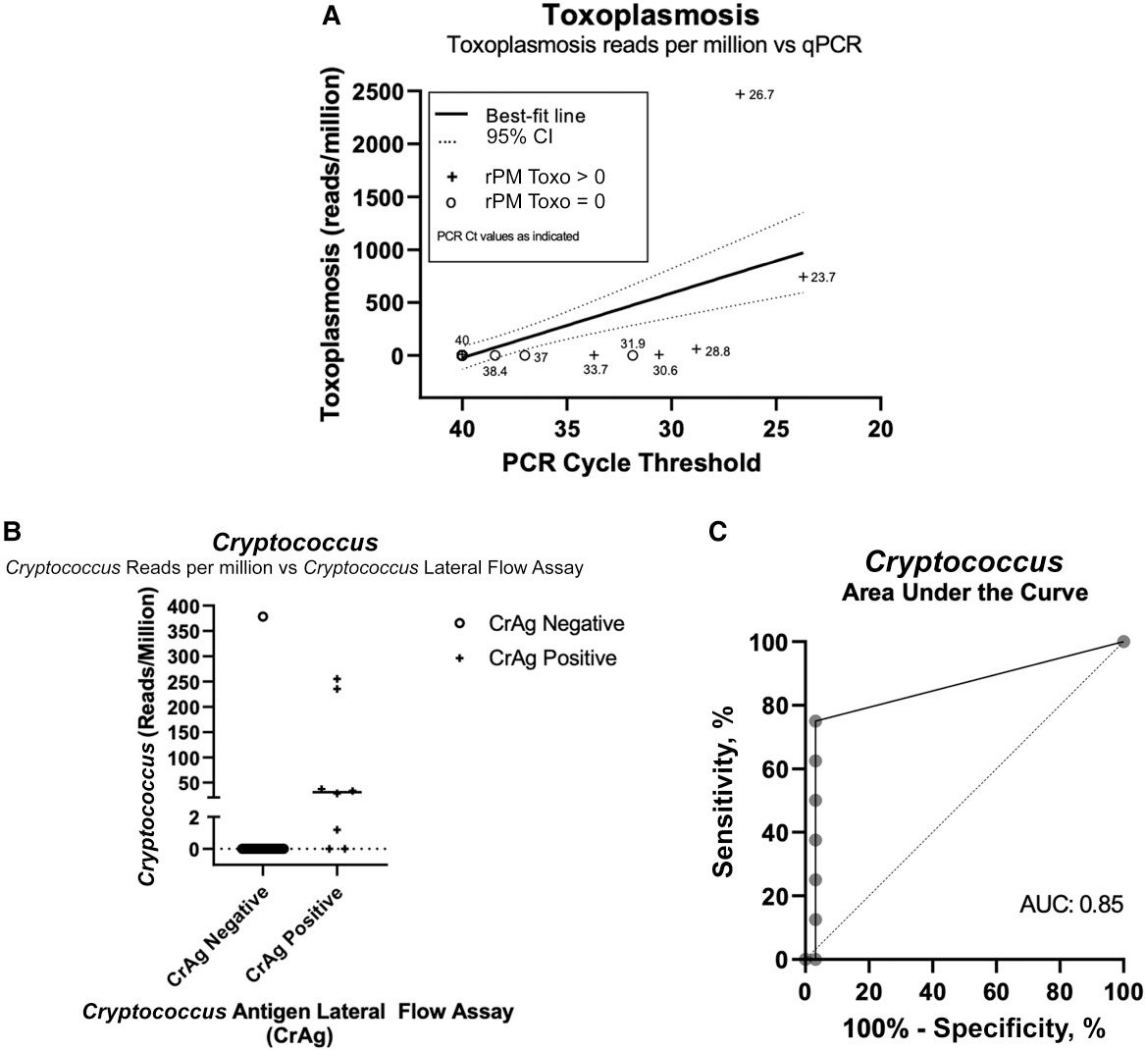
Relationship between metagenomic next-generation sequencing (mNGS) and known clinical measures of disease. *A*, Comparison of mNGS reads per million for *Toxoplasma gondii* to *T gondii* quantitative polymerase chain reaction cycle threshold in cerebrospinal fluid. *B*, Comparison of mNGS reads per million for *Cryptococcus neoformans* to cryptococcal antigen lateral flow assay (CrAg-LFA) results. *C*, Analysis of area under the curve for mNGS as compared to CrAg-LFA results. Abbreviations: AUC, area under the curve; CI, confidence interval; CrAg, cryptococcal antigen; LFA, lateral flow assay; PCR, polymerase chain reaction; qPCR, quantitative polymerase chain reaction; rPM, reads Per Million.

mNGS detected *T gondii* sequences in 5 of the 7 *T gondii* qPCR-positive samples and in an additional 3 qPCR-negative samples. Head CT findings were suggestive of possible cerebral toxoplasmosis in 1 of 3 discordant participants. There was a negative correlation between toxoplasmosis qPCR Ct and the number of mNGS reads mapping to *T gondii* (Figure 3*A*). Positive percentage agreement between *T gondii* qPCR and mNGS was 71.4% (95% CI, 29%–96.3%) and negative percentage agreement was 91.4% (95% CI, 76.9%–98.2%) (Table 6). Reanalysis of clinical covariates after inclusion of mNGS *T gondii*–positive participants did not change findings (data not shown).

**Table 6.** Diagnostic Agreement of *Toxoplasma gondii* Quantitative Polymerase Chain Reaction and Metagenomic Next-Generation Sequencing

		qPCR		
Assay		Positive	Negative	Total
mNGS	Positive	5	3	8
	Negative	2	32	34
	Total	7	35	42
Probability (95% CI)				
Positive percentage agreement	71.4% (29%–96.3%)			
Negative percentage agreement	91.4% (76.9%–98.2%)			

Abbreviations: CI, confidence interval; mNGS, metagenomic next-generation sequencing; qPCR, quantitative polymerase chain reaction.

Of 9 CrAg-LFA positive samples sequenced, mNGS detected *C neoformans* sequences in 7 (78%). The 2 CrAg-LFA positive, mNGS-negative samples had head CTs consistent with meningitis. Comparison of mNGS results to CrAg-LFA reveal an area under the curve of 0.89 (95% CI, .745–1), sensitivity of 78% (95% CI, 40%–97%), and specificity of 100% (95% CI, 88%–99.9%) (Table 7, Figure 3*B* and 3*C*). One sample that contained high abundance of *C neoformans* sequences by mNGS was initially negative by CrAg-LFA; subsequently, the specimen was serially diluted and retested by CrAg-LFA with a positive result. CrAg-LFA has a known prozone effect (where high antigen concentration impedes antibody sandwich formation in 1-step immunoassays), and the test manual suggests that users serially dilute specimens when high antigen concentration is suspected [13, 14].

**Table 7.** Sensitivity and Specificity of Cerebrospinal Fluid Metagenomic Next-Generation Sequencing Versus Cryptococcal Antigen Lateral Flow Assay

		CrAg (Serum or CSF)		
Assay		Positive	Negative	Total
CSF mNGS	Positive	7	0	7
	Negative	2	30	30
	Total	9	30	39
Probability (95% CI)				
Area under the curve	0.89 (.745–1)			
Sensitivity	78% (40%–97%)			
Specificity	100% (88%–99.9%)			

Abbreviations: CI, confidence interval; CrAg, cryptococcal antigen; CSF, cerebrospinal fluid; mNGS, metagenomic next-generation sequencing.

mNGS identified *M tuberculosis* sequences in 5 participants’ CSF. Noncontrast head CTs were available for 4 of these participants, and none of the CT reports suggested tuberculous meningitis (TBM) as a likely diagnosis. Two participants with suspected tuberculosis (TB) had no respiratory symptoms or examination findings, history of TB, or known TB contacts. Three participants reported nonbloody productive cough for >2 weeks. Three of 5 participants with TB detected by mNGS had a presumptive diagnosis of TBM and were treated; 2 survived.

One sample with TB also contained HBV DNA, and 1 sample contained pegivirus RNA. Pegiviruses (Flaviviridae) are usually considered nonpathogenic to humans, and HBV detection was likely unrelated to the participant's neurologic condition and may represent contamination with peripheral blood [15]. The participants with HBV and pegivirus had normal abdominal examinations and no scleral icterus. Nine samples contained Epstein-Barr virus and 2 contained cytomegalovirus. The average rPM was 0.7. None of the cases met the predefined threshold to be considered a potential pathogen.

## DISCUSSION

We describe clinical features of PWH presenting with acute neurological conditions from Iquitos, Peru, focusing on cerebral toxoplasmosis and cryptococcal meningitis. We further show that mNGS has potential to increase diagnostic yield of these infections as well as identify other, sometimes unanticipated, pathogens.

Prevalence of cerebral toxoplasmosis in this population was 24% by CSF qPCR. Though not classified as cases in our analyses, 3 additional participants were diagnosed with cerebral toxoplasmosis by CSF mNGS, bringing prevalence to 26%. These results corroborate other studies of PWH conducted in the Amazon region [16, 17], where *T gondii* seroprevalence is very high (97% in this study). As in other studies, participants with cerebral toxoplasmosis in this study generally had nonspecific presentations, including unilateral focal motor signs and positive Babinski reflex [1, 18, 19]. Almost all (17/18) *T gondii* qPCR-positive patients who underwent head CT had focal lesions. The direct correlation between number of lesions on head CT and parasite load suggests that intracranial lesions are associated with more severe disease. Several participants with negative qPCR had head CT findings suspicious for toxoplasmosis. However, the suboptimal head CT conditions (majority lacked contrast and follow-up CT for signs of clinical improvement after initiation of treatment) led us to include a CT result as supportive of a diagnosis but not as a diagnostic criterion itself.

The prevalence of cryptococcal meningitis was 11% in our study, slightly higher than in other reports, though studies from the Amazon region are lacking [20–22]. All 13 participants with cryptococcal meningitis reported headache and weight loss, and most reported photophobia. Headache and photophobia are common symptoms of cryptococcal meningitis; weight loss, however, is less frequently identified but may reflect advanced HIV [1, 23]. This study defined participants who were CrAg positive in CSF and/or serum as probable cases of cryptococcal meningitis. This decision was based on our internal data demonstrating a κ = 1 between serum and CSF CrAg in participants who received both assays and on prior work from Williams et al, which revealed that 93% of serum CrAg-positive patients also had a positive CSF CrAg assay [24].

Six-month mortality in this population was very high at 54%. Forty percent of study participants were diagnosed with HIV within the 3 months prior to enrollment with low CD4 counts (median CD4 count of 119 cells/mm^3^), which likely was a key factor in the high mortality. Previous work in Loreto has shown high rates of discrimination against PWH and misconceptions about HIV, creating barriers to testing, care, and treatment adherence [25]. Though 52% of participants were on ARVs, a third of those forgot to take their medication ≥3 times per week.

PWH are susceptible to (and frequently coinfected with) many organisms, so techniques that allow simultaneous testing for multiple pathogens offer an attractive alternative to multiple assays for individual pathogens. Multiplex PCR technology and next-generation sequencing both represent great advancements for this purpose. The commercially available BioFire assay shows great promise as a multiplex assay but is limited by the available targeted organisms (which currently only include 1 parasitic infection). However, it has equipment that is relatively portable. mNGS resolves the limitation of a priori pathogen selection but is not portable. Our comparison of mNGS to *T gondii* qPCR and CrAg demonstrated that although mNGS is helpful to diagnose central nervous system OIs, it can be less sensitive than optimized pathogen-specific techniques, especially for pathogens at low abundance in CSF [26, 27]. However, limited sensitivity of *T gondii* qPCR confounds estimates of mNGS performance to diagnose cerebral toxoplasmosis; indeed, mNGS identified 3 additional possible cerebral toxoplasmosis cases that were qPCR negative. Because of *T gondii* qPCR's limited sensitivity, we reported percentage agreement with mNGS instead of sensitivity and specificity. However, the CrAg assay has excellent sensitivity and can serve as a gold standard to estimate the sensitivity and specificity of mNGS for cryptococcal meningitis. While the CrAg-LFA is reported to have 100% sensitivity and specificity, a prozone phenomenon has been reported [28], as we documented in 1 sample in our study. Finally, mNGS identified TB DNA in 5 participants, only 3 of whom had been treated for TBM, highlighting its ability to identify unsuspected infections [11]. mNGS may be useful in TB-endemic regions, as qPCR and even GeneXpert have limited sensitivity in CSF, and culture can take weeks. The identification of HBV and pegivirus sequences in 2 samples demonstrate the ability of mNGS to identify infections incidental to a patient's neurological presentation and the need for clinical adjudication [29].

This study had several limitations. Sample size limited our power to detect risk factors for infection with *T gondii* and *C neoformans*. CSF was not obtained from all patients due to mass lesions, patient/physician preference, or early death, and sufficient CSF was only available to perform mNGS on a subset of samples. Basic data about the CSF were not available (cells, opening pressure). *Toxoplasma gondii* qPCR has limited sensitivity (35%–70%) but is the best available nonsurgical test; it is probable that more patients in our cohort had cerebral toxoplasmosis, including those identified by mNGS [4–6]. Because of CrAg-LFA supply chain challenges, the first 86 participants were tested retrospectively for *C neoformans* using stored samples that may have degraded over time. Head CTs were only available for 43% of participants, and only 16% of those were performed with intravenous contrast; in some cases, this was due to medical contraindications but more frequently to lack of contrast at the study site. Additionally, we could not use change in CT findings to diagnose cerebral toxoplasmosis because only 8 posttreatment CTs were obtained. Mortality data were obtained through retrospective chart review 6 months after study completion, and dates of death were not available. Detailed information about treatment dose and duration for both infections was unavailable, preventing evaluation of prophylaxis or treatment on outcomes.

Despite these limitations, this study offers insight into the epidemiology and clinical features of common neurological OIs and into shortcomings of diagnostic tests. Both cerebral toxoplasmosis and latent infection were highly prevalent in our study population. The nonspecific presentation of cerebral toxoplasmosis, imperfect sensitivity of available laboratory assays, and limited availability of biopsy underscore the need for improved diagnostic testing. While CrAg-LFA performs much better than toxoplasmosis diagnostics, our mNGS results revealed limitations to this test as well. Our exploration of a genomic avenue for diagnosis yielded hopeful results that call for further development. Unfortunately, even if these novel techniques achieve the necessary sensitivity and specificity, access to and expertise in nontargeted sequencing techniques is currently limited in resource-limited areas. Thus, ideal next steps are improvements in access to point-of-care testing and treatment, for both HIV and their associated OIs, to decrease the burden of disease and high mortality.

## Supplementary Material

ofad515_Supplementary_Data

## Supplementary Data

Supplementary materials are available at *Open Forum Infectious Diseases* online. Consisting of data provided by the authors to benefit the reader, the posted materials are not copyedited and are the sole responsibility of the authors, so questions or comments should be addressed to the corresponding author.

## Notes

***Acknowledgments*.** The Toxoplasmosis Working Group in Peru: Catherine Apaza, Melanie Ayachi, Oliver A. Bocanegra, Jeroen Bok, Linda Chanamé Pinedo, Marilly Donayre Urquizo, Renzo Gutierrez-Loli, Gaston Pinedo, Grace Trompeter, Sory Vazquez, and Deanna Zhu.

***Author contributions.*** H. E. S., C. F., M. S., and N. M. B. contributed to the statistical analysis. P. S. R., A. W., K. C. Z., and M. R. W. contributed to the next-generation sequencing and analysis. M. M. D. read all computed tomography scans. A. D. and M. C. completed all of the *T gondii* qPCR assays. L. P., A. D., and M. C. completed all the CrAg-LFAs. H. E. S., L. P., D. E. K., L. C., V. P.-C., C. R. A., R. H. G., and N. M. B. all assisted with study management, patient recruitment, and/or specimen processing. All authors contributed to the writing and editing of the manuscript.

***Financial support*.** This work was supported by the National Institutes of Health (grant/award numbers K23 AI113197 to N. M. B., D43TW009340 to H. E. S., and R01Al136722-01A1 and D43TW010074-04 to R. H. G.); Innóvate Peru (grant number 137-PNICP-PIAP-2015 to M. C.); the Burroughs-Wellcome Fund/American Society of Tropical Medicine and Hygiene Postdoctoral Fellowship in Tropical Infectious Diseases (to N. M. B.); Neurology Clinical Research Training Scholarship (award number P0534134 to P. S. R.); Australian Government Research Training Program Scholarship (to P. S. R.); Westridge Foundation (to M. R. W.); and Medical Research Council Clinical Research Training Fellowship (award number MR/P019978/2 to D. E. K.). University funding was provided to the University of North Carolina Center for AIDS Research (grant number P30 AI050410 to N. M. B.); Dean’s Biomedical Sciences Scholar Award, University of Illinois Chicago (to H. E. S.); Universidad Peruana Cayetano Heredia (to M. C.); and National University of San Marcos (to M. C.).
