# Supplementary material for: Clinical and Metagenomic Characterization of Neurological Infections of People With Human Immunodeficiency Virus in the Peruvian Amazon
Source: Open Forum Infect Dis. 2023 Oct 27;10(11):ofad515. doi: 10.1093/ofid/ofad515 (PMC10642733; doi:10.1093/ofid/ofad515)
Supplement: ofad515_Supplementary_Data [file ofad515_supplementary_data.docx]

**Methods:**

*Study procedures:*

Upon entry, study staff used a questionnaire to elicit demographic, travel, and medical histories and risk factors for HIV from participants, or their health care proxy if their neurological status was impaired. A full clinical exam followed including mini-mental status exam and HIV dementia scale.^16^ Blood and urine specimens were collected at the time of enrollment. Blood was drawn by a qualified study nurse or doctor. Remnant CSF was given to the study if a lumbar puncture was ordered by the treating clinician and additional CSF was available after clinical testing. CSF specimens were either placed immediately at -20˚C and transferred to a -80˚C freezer at the end of the week or transported on wet ice to a -80˚C freezer approximately seven kilometers from the hospital. Blood specimens collected in a red top tube were spun for 10 min at 3000RPM, serum was separated from the clot and both components frozen. Complete blood count, CD4 count, CD8 count, and HIV viral loads were abstracted from the patient’s chart.

*Specific pathogen testing*

*C. neoformans* antigen testing (Immy, Norman Oklahoma, CrAg LFA): Patients’ serum samples were first tested, and if positive, CSF was tested when available for secondary confirmation. Serum samples were tested per manufacturer’s instructions. Briefly, one drop of lateral flow specimen diluent was placed in a microfuge tube. 40uL of serum was added to the diluent. The CrAg test strip was placed in the microfuge tube, and the results read after an interval of 10 minutes to two hours. If the serum test was positive and CSF from the patient was available, the same procedure was repeated with CSF.

qPCR for *T. gondii*: qPCR of blood clot and CSF samples were performed as previously described.^17,18^ Six molar guanidine hydrocholoride was added to samples prior to homogenization with a homogenizer. Homogenized samples were lysed using a Matrix H 2 mL tube (MP Biomedicals, USA). Lysed samples were agitated in a FastPrep-24™ 5G machine (MP Biomedicals, USA) (5.5 m/s – 30”). Parasite DNA was extracted using Roche Life Sciences’ (Basal, Switzerland) High Pure PCR Template Preparation Kit (Catalog Number 11796828001). Primers to *T. gondii*’s repeat 529 were used 5′- GCTCCTCCAGCCGTCTTG (forward) and 5′- TCCTCACCCTCGCCTTCAT (reverse), and FAM - AGGAGAGATATCAGGACTGTA - 3'MGB probe.^18,19^ A thermocycler (applied biosystems) was used to amplify the DNA with 2 minutes at 50˚C, 10 minutes at 95˚C; and 40 cycles of 95˚C for 15 seconds, 60 seconds at 58˚C, and 60 seconds at 72˚C; followed by a 4˚C hold.

The positive cycle threshold cut-off for each plate was established based on the parasite standard curve. For a specimen to be considered positive by qPCR, it needed to have a cycle threshold less than the highest cycle threshold on the standard curve, equivalent to the DNA of one tachyzoite (highest cycle threshold cut-off in the study=37). Each sample was analyzed by qPCR twice. Both replicates needed to be positive for the specimen to be considered positive.

*T. gondii* serology: IgG serology was assessed by in-house enzyme linked immunosorbent assay (ELISA). Tachyzoite lysate antigen was prepared as previously described ^17,18,20^. Briefly, eight day monkey kidney fibroblast (LLC-MK_2_) tachyzoite co-cultures were lysed with three freeze thaw cycles, four cycles on sonification (4Hz for 30s each, with 1 minute rest between cycles), and a final centrifugation (10,000g for 20 minutes at 4˚C) after which the supernatant was collected and evaluated for total protein concentration by the Bradford method. Tachyzoite lysate antigen (100uL from a 1ug/mL stock) was absorbed onto Nunc MaxiSorp 96 well ELISA plates (Nunc Nalgene, Rochester, NY) overnight at 4˚C. Plates were washed with phosphate buffered saline (PBS)-tween 20 (0.05%). Plates were then blocked with 5% dried skim milk, suspended in PBS-tween 20 (0.05%) for 1 hour. Blocked plates were then loaded with patient serum samples, diluted 1:500, and incubated for 1 hour at 37˚C. Plates were washed with PBS-Tween 20 (0.05%). Horseradish Peroxidase goat anti-human IgG antibody (SeraCare Life Sciences, Milford, MA) was diluted 1:10000, 100uL were added to each well, and the plate was incubated for 1 hour at 37˚C. 3,3’,5,5’-tetramethylbenzidine (TMB) substrate (100 uL/well) (Thermo Fisher Scientific, Waltham, MA) was incubated for 5 min in the dark at RT. The reaction was stopped using 50 ul of 2M H_2_SO_4_. The optical density (OD) of each well was evaluated at 450nM with a plate reader (Molecular Devices, LLC, San Jose, CA). Specimens were considered positive if their OD was greater than three times the standard deviation of the mean absorbance of the negative controls. The in-house ELISA was evaluated against IBL international’s *T. gondii* IgG ELISA kit. Evaluation consisted of 38 serum samples, 19 non-clinical samples and 19 PLHIV. Samples were tested in duplicate to assess reproducibility. In-house ELISA had a sensitivity of 100% (95%CI: 83-100%), a specificity of 92.3% (95%CI:64-99%), and a kappa index of 0.94 (95%CI: 0.83-1.06), with a concordance of 0.97.^20^

*CSF Metagenomic Next-Generation Sequencing*

Total nucleic acid was extracted from 90 uL of CSF using the Zymo *Quick*-DNA/RNA MagBead (Zymo Cat. No. R2130) via the Integra Viaflo 96 as per the manufacturer’s protocol. No additional steps such as bead washing or sonication were taken. RNA sequencing (RNA-Seq) libraries were prepared using the New England Biolabs’ NEBNext Ultra II RNA library preparation kit (NEB Cat No. E7770) and DNA libraries (DNA-seq) were prepared using the New England Biolabs’ NEBNext® Ultra™ II FS DNA Library Prep Kit (E7805L), as per the protocol. Library preparation was performed in bulk using the Echo Labcyte 525 and Integra Viaflo 96 liquid handling robots. Host ribosomal RNA depletion was performed using the Qiagen QIAseq FastSelect RNA removal kit (Qiagen Cat No. 333180) at 1:100 dilution. An open source, cloud-based metagenomics pipeline CZID was used for mNGS analysis.^21^

**Supplemental Table 1: Results of *T. gondii* qPCR, CrAg, mNGS and Head CT findings for all patients who underwent head CT**

| **Patient Number** | **Toxoplasmosis PCR in CSF** | **CrAg CSF or Serum** | **NGS** | **Head CT Findings** | | |
| --- | --- | --- | --- | --- | --- | --- |
|  |  |  |  | **Probable** | **Possible** | **Less Likely** |
| 11 | Negative |  |  | TB* | NCC*, Crypto* | Toxo* |
| 20 | Negative |  |  | Toxo | TB |  |
| 21 | Negative |  |  | TB |  | Crypto, Toxo |
| 22 | Negative |  |  | TB | Toxo |  |
| 23 | Positive |  |  | Toxo |  | Crypto, TB |
| 27 | Negative |  |  | Normal |  |  |
| 28 | Positive |  | Negative | Normal |  | Toxo, TB, Crypto, viral encephalitis, malaria. |
| 30 | Positive |  |  |  | Toxo | Crypto, TB, NCC |
| 33 | Negative |  |  | Normal |  | Toxo, TB, viral encephalitis, malaria |
| 35 |  | Negative (S)* |  | NCC |  |  |
| 38 |  | Negative (S) |  | Normal |  |  |
| 39 |  | Positive (S) |  | Toxo, TB |  |  |
| 40 | Negative | Positive (S) |  | Normal |  |  |
| 41 | Negative | Negative (S) |  | TB |  | Toxo |
| 43 | Positive | Negative (S) |  |  |  | Toxo, TB |
| 45 |  | Negative (S) |  | Toxo | TB |  |
| 46 |  | Negative (S) |  | Toxo |  |  |
| 47 | Negative | Negative (S) | Negative | Meningoencephalitis (Crypto, TB, viral) |  |  |
| 49 | Negative | Positive (S) | Toxo | Meningoencephalitis (Crypto, TB) |  |  |
| 50 | Negative | Negative (S) |  | TB |  |  |
| 52 |  | Positive (S) | Negative | Meningoencephalitis (Crypto, TB) |  |  |
| 53 |  | Negative (S) |  | TB |  | Toxo |
| 54 |  | Negative (S) |  | Normal |  |  |
| 55 |  | Negative (S) |  | Meningoencephalitis (Crypto, TB) |  |  |
| 56 | Negative | Negative (S) |  | Meningoencephalitis (TB) |  |  |
| 57 |  | Negative (S) |  | TB | Crypto, Toxo |  |
| 58 | Negative |  |  | Meningoencephalitis (Viral, TB, Malaria) |  |  |
| 59 | Positive | Negative (S) | Toxo | Toxo |  | TB |
| 60 |  | Negative (S) |  | Meningoencephalitis (Viral, TB, Malaria) |  |  |
| 61 | Negative | Negative (S) | Negative | Meningoencephalitis (Viral, TB, Malaria) |  |  |
| 62 | Negative | Negative (S) | Negative | Unknown |  | Toxo, TB |
| 63 | Negative | Negative (S) | Negative | IRIS |  |  |
| 64 | Positive | Negative (S) | Toxo |  | PCNSL, Toxo |  |
| 65 | Negative | Positive (S) | Crypto | Meningoencephalitis (Crypto, TB, Viral, Malaria) |  |  |
| 66 | Negative | Negative (S) | Negative |  | Crypto, TB | Toxo |
| 67 | Negative | Negative (S) | Toxo | PML | Toxo |  |
| 68 | Negative | Negative (S) | TB and Hepatitis B |  | Toxo | Crypto |
| 69 | Negative | Negative (S) | Negative | Toxo |  | Crypto, TB |
| 70 | Negative | Negative (S) | Negative | Metastatic Lesions |  | Crypto, Toxo, TB |
| 71 | Positive | Negative (S) | Negative |  | Toxo, TB |  |
| 72 | Negative | Negative (S) |  | Unknown |  |  |
| 73 |  | Negative (S) |  | Toxo, PCNSL |  |  |
| 75 | Negative | Negative (S) |  | Crypto | TB |  |
| 76 |  | Negative (S) |  | Toxo | Crypto |  |
| 77 | Positive | Negative (S) |  | Toxo |  |  |
| 78 | Negative | Negative (S) |  | Unknown |  |  |
| 79 |  | Negative (S) |  |  | Toxo | NCC |
| 80 | Negative | Negative (S) | TB | Unknown |  |  |
| 81 | Positive | Negative (S) | Toxo | TB |  | Crypto, Toxo |
| 84 | Positive | Negative (S) |  | TB |  | Crypto, Toxo |
| 85 | Positive | Negative (S) |  | Meningioma |  |  |
| 86 | Negative | Positive (C)* | Crypto | TB | Crypto |  |
| 87 | Negative | Negative (S) |  | TB | Crypto |  |
| 88 | Negative |  |  |  |  | Toxo, Crypto, TB, PML |
| 91 | Negative | Positive (C) | Crypto | Meningoencephalitis (Crypto, TB, Viral) |  |  |
| 93 | Positive |  | Negative | Meningoencephalitis (Viral, TB) |  |  |
| 94 | Negative |  |  | Normal |  |  |
| 122 | Negative |  |  | Normal |  |  |
| 124 | Negative | Negative (C) | Negative | Toxo | TB, PML |  |
| 126 | Positive | Negative (C) | Toxo | Toxo |  | Crypto, TB |
| 131 | Negative | Negative (C) |  | Normal |  |  |
| 132 |  | Negative |  | Toxo |  |  |
| 133 | Negative | Negative (C) |  | Unknown |  |  |
| 135 | Negative | Negative (C) |  | Infarct |  |  |
| 137 | Negative | Negative (C) |  | Unknown |  |  |
| 138 | Negative | Negative (C) |  | PCNSL | Infarct |  |
| 139 | Positive | Negative (C) |  | Toxo |  |  |
| 140 | Negative | Negative |  | Normal |  |  |
| 141 | Negative | Negative (C) | Negative | Infarct |  |  |
| 142 | Negative | Negative (C) | Negative | Normal |  |  |
| 143 | Negative | Negative (C) |  | Normal |  |  |
| 144 | Negative | Negative (C) |  | Toxo |  | Crypto |
| 146 | Positive | Negative (C) |  | Toxo |  |  |
| 147 | Negative | Negative (C) |  | Toxo |  | Crypto, TB |
| 148 | Negative | Negative (C) | Pegivirus | Normal |  |  |
| 149 | Positive | Negative (C) |  | Toxo | TB |  |
| 150 | Negative | Negative (C) |  | Normal |  |  |
| 151 | Negative | Negative (C) |  | Normal |  |  |
| 152 | Negative | Negative (C) |  | Infarct |  |  |
| 153 | Negative | Negative (C) |  | Unknown |  |  |
| 154 | Negative | Negative (C) |  | Toxo |  |  |
| 155 | Negative | Positive (C) | Crypto | Normal |  |  |
| 156 | Negative | Negative (C) |  | Normal |  |  |
| 157 | Negative | Negative (C) | TB | Normal |  |  |
| 158 | Positive | Negative (C) |  | PCNSL |  |  |
| 159 | Negative | Negative (C) | TB | Normal |  |  |
| 160 | Positive | Negative (C) |  | Toxo | TB, Crypto |  |
| 161 | Negative | Negative (C) | Negative | Unknown |  |  |
| 162 | Negative | Negative (C) | Negative | Normal |  |  |
| 163 |  | Negative (C) |  | Toxo | Crypto, TB, Chagas |  |
| 164 |  | Negative (C) |  |  | Crypto, TB |  |
| 165 | Positive | Negative (C) |  | Toxo |  |  |
| 166 | Negative | Positive (C) |  | Infarct |  |  |
| 167 |  | Negative (C) |  |  | Meningoencephalitis (Crypto, TB) |  |
| 168 | Negative | Negative (C) |  | Unknown |  |  |
| 169 | Positive | Negative (C) |  | Toxo |  |  |
| *Abbreviations: Crypto: Cryptococcus, NCC: Neurocysticercosis, Toxo: Toxoplasmosis, TB: Tuberculosis, (C): CSF, (S): serum | | | | | | |

**Supplemental Table 2: Comparison of CT Findings With and Without contrast among patients tested by qPCR for *Toxoplasma gondii* in CSF**

|  | | qPCR  Positive N=6 | qPCR  Negative N=8 | p-value* | qPCR  Positive N=12 | | qPCR  Negative N=52 | p-value* |  |
| --- | --- | --- | --- | --- | --- | --- | --- | --- | --- |
|  |  | N (%) | N (%) |  | N (%) | | N (%) |  |  |
|  |  | **Computed Tomography Findings with Contrast** | | | | **Computed Tomography Findings Without Contrast** | | | |
| Lesions (% Yes) | | 6/6 (100%) | 2/8 (25%) | **0.007** | 10/12 (83%) | | 27/52 (52%) | **0.05** |  |
| Number of Lesions | | | | | | | | | |
|  | None | 0/6 (0%) | 6/8 (75%) | **0.026** | 2/12 (17%) | | 26/52 (50%) | **0.009** |  |
|  | 1 | 2/6 (33%) | 0/8 (0%) |  | 3/12 (25%) | | 13/52 (25%) |  |  |
|  | 2-5 | 2/6 (33%) | 1/8 (12.5%) |  | 4/12 (33%) | | 11/52 (21%) |  |  |
|  | 6-10 | 1/6 (17%%) | 1/8 (12.5%) |  | 2/12 (17%) | | 2/52 (4%) |  |  |
|  | 11-20 | 1/6 (17%) | 0/12 (0%) |  | 1/12 (8%) | | 0/52 (0%) |  |  |
| Number Hyperdense Lesions | | | | | | | | | |
|  | None | 3/6 (50%) | 8/8 (100%) | **0.031** | 5/12 (42%) | | 46/52 (88%) | **0.0003** |  |
|  | 1 | 1/6 (17%) | 0/8 (0%) |  | 4/12 (33%) | | 4/52 (8%) |  |  |
|  | 2-5 | 2/6 (33%) | 0/8 (0%) |  | 2/12 (17%) | | 2/52 (4%) |  |  |
|  | 6-10 | 0/6 (0%) | 0/8 (0%) |  | 0/12 (0%) | | 0/52 (0%) |  |  |
|  | 11-20 | 0/6 (0%) | 0/8 (0%) |  | 1/12 (8%) | | 0/52 (0%) |  |  |
| Number Hypodense Lesions | | | | | | | | | |
|  | None | 1/6 (17%%) | 6/8 (75%) | 0.061 | 6/12 (50%) | | 29/52 (56%) | 0.398 |  |
|  | 1 | 1/6 (17%%) | 0/8 (0%) |  | 1/12 (8%) | | 10/52 (19%) |  |  |
|  | 2-5 | 2/6 (33%) | 1/8 (12.5%) |  | 3/12 (25%) | | 11/52 (21%) |  |  |
|  | 6-10 | 1/6 (17%%) | 1/8 (12.5%) |  | 2/12 (17%) | | 2/52 (4%) |  |  |
|  | 11-20 | 1/6 (17%%) | 0/8 (0%) |  | 0/12 (0%) | | 0/8 (0%) |  |  |
| Number Affected Hemispheres | | | | | | | | | |
|  | None | 0/6 (0%) | 6/8 (75%) | **0.013** | 3/12 (25%) | | 25/52 (48%) | **0.047** |  |
|  | One | 3/6 (50%) | 1/8 (12.5%) |  | 3/12 (25%) | | 17/52 (33%) |  |  |
|  | Two | 3/6 (50%) | 1/8 (12.5%) |  | 6/12 (50%) | | 10/52 (19%) |  |  |
| Edema | | | | | | | | | |
|  | None | 0/6 (0%) | 7/8 (87.5%) | **0.023** | 3/12 (25%) | | 31/52 (60%) | **0.013** |  |
|  | Minor | 0/6 (0%) | 1/8 (12.5%) |  | 5/12 (42%) | | 16/52 (31%) |  |  |
|  | Moderate | 5/6 (83%) | 0/8 (0%) |  | 2/12 (17%) | | 4/52 (8%) |  |  |
|  | Severe | 1/6 (17%) | 0/8 (0%) |  | 2/12 (17%) | | 1/52 (2%) |  |  |
| Mass Effect | | | | | | | | | |
|  | None | 0/6 (0%) | 8/8 (100%) | **0.019** | 6/12 (50%) | | 44/52 (85%) | **0.007** |  |
|  | Minor | 2/6 (33%) | 0/8 (0%) |  | 3/12 (25%) | | 6/52 (12%) |  |  |
|  | Moderate | 3/6 (50%) | 0/8 (0%) |  | 3/12 (25%) | | 2/52 (4%) |  |  |
|  | Severe | 1/6 (17%) | 0/8 (0%) |  | 0/12 (0%) | | 0/52 (0%) |  |  |
| Hydrocephalus (%Yes) | | 1/6 (17%) | 1/8 (12.5%) | 0.897 | 0/12 (0%) | | 4/52 (8%) | 0.325 |  |
| White Matter Changes (%Yes) | | 4/6 (67%) | 0/8 (0%) | **0.009** | 5/12 (42%) | | 12/52 (23%) | 0.192 |  |
| Type of White Matter Changes | | | | | | | | | |
|  | None | 2/6 (33%) | 8/8 (100%) | **0.009** | 7/12 (58%) | | 40/52 (77%) | 0.178 |  |
|  | Minor | 0/6 (0%) | 0/8 (0%) |  | 3/12 (25%) | | 9/52 (17%) |  |  |
|  | Moderate | 4/6 (67%) | 0/8 (0%) |  | 2/12 (17%) | | 1/52 (2%) |  |  |
|  | Generalized | 0/6 (0%) | 0/8 (0%) |  | 0/12 (0%) | | 2/52 (4%) |  |  |
| *p-values were calculated with Kruskal–Wallis | | | | | | | | | |

| Supplemental Table 3: Participants with Follow up CTs | | | | | |
| --- | --- | --- | --- | --- | --- |
| Patient Code | qPCR Toxoplasmosis | CrAg Cryptococcus | Initial Summary | Days since initial CT | Repeat CT Summary |
| 21 | Negative | Not Tested | 36M, HIV, CD4 135, VL detectable, hx treated TB, p/w seizures, headaches. CT Interpretation: contrasted CT, no e/o pachy- or leptomeningeal enhancement, normal CT scan. Likely tuberculous meningoencephalitis given hx, crypto less likely with CD4; toxo less likely no lesions discernible. | 7 | contrasted CT, no e/o pachy- or leptomeningeal enhancement, normal CT scan. Likely tuberculous meningoencephalitis given hx, crypto less likely with CD4; toxo less likely since no lesions discernible. |
| 23 | Positive | Not Tested | 37M HIV (unknown CD4 and VL), p/w fever, weight loss, headaches (no sz). CT Interpretation: confluent hypodensities representing diffuse vasogenic edema involving R occipital, b/l parietal, b/l frontal lobes; mild mass effect into R lateral ventricle. Contrasted CT reveals partial ring enhancing lesion in R internal capsule, possible ring enhancing lesion in R BG. Most likely CNS toxo; cryptococomas or TB less likely with this appearance. | 369 | several small hypodensities involving b/l basal ganglias; diffuse vasogenic edema from 2016 CT has resolved. |
| 54 | No Specimen | Negative | Male recent dx HIV, CD4 unknown, p/w extrapyramidal symptoms, seizures, headaches. Scan 1: CT head normal; somewhat motion limited. | 7 | New hypodensity in right basal ganglia not present on CT 1 week prior (extending from caudate to putamen), minimal vasogenic edema. Consider CNS toxoplamsosis or crypto lesion given evolution. |
| 62 | Negative | Negative | 37M HIV (unknown CD4) p/w fevers, weight loss, no headaches, no seizures, +TBC hx, GCS 14, +focal neuro exam Scan 1: CT interpretation: L basal ganglia faint nodular hyperdensity with mild surrounding vasogenic edema; 1 small hypodensity in R frontal white matter. CT findings non-specific, given basal ganglia location could represent CNS toxo or CNS TB, crypto. | 39 | CT unchanged from prior 1 month ago. Left BG hyperdense nodular lesion with mild vasogenic edema still present, and R frontal hypodensity still present. Unclear if toxo lesions improved with treatment without contrasted CT |
| 73 | No Specimen | Negative | 28M HIV (unknown CD4) p/w fevers, seizures, headaches, AMS, GCS 11, focal motor deficit no hx TB, Scan1: Left thalamic nodular lesion with hyperdense core likely calcification and massive vasogenic edema throughout frontal lobe extending into left midbrain and R basal ganglia, with midline shift, mass effect into contralateral ventricle, no uncal herniation. R basal ganglia hypodensity does not seem to be contiguous with left side, could represent second lesion. Bsaed on appearance this is toxo, or possible PCNSL. | 16 | Markedly improved vasogenic edema, minimal midline shift and mass effect. Hyperdense lesion appears smaller, less extensive. Given marked improvement with toxo treatment, most likely CNS Toxo |
| 135 | Negative | Negative | >1cm hypodensity involving the right parieto-occipital lobe, given its appearance this is a subacute ischemic infarct involving right parieto-occipital lobe | 87 | Evolving ischemic infarct involving right parieto-occipital lobethat now appears chronic on this CT scan compared with Jan 2019 CT |
| 137 | Negative | Negative | Communicating hydrocephalus present, possible small hypodensity in the left frontal lobe but this appears more to be artifact | 24 | Bifrontal periventricular hypodensities (R>L frontal lobes) and L thalamic nodular appearing hypodensity without mass effect; moderate communicating hydrocephalus; given location of hypodensities could be CNS TB vs cryptococcoma vs possible toxo given thalamic location but difficult to tell without contrast |
| 160 | Positive | Negative | Multiple nodular appearing lesions with hyperdensity in center of each lesion; largest lesion is in the right basal ganglia with edema, mass effect and midline shift; given location and edema, likely CNS toxoplasmosis, could also be CNS TB vs cryptococcomas as well but suspect CNS toxo given intralesional hyperdensities | 16 | Interval resolution of mass effect and midline shift seen on previous CT, hyperdensity in right basal ganglia improved; likely reflects response to treatment; still suspect CNS Toxoplasmosis based on resolution of peri-lesional edema with presumed treatment over 1 month |

**Supplemental Table 4: Relationship between CrAg results in patient serum and CSF**

|  | | CSF | | |
| --- | --- | --- | --- | --- |
|  |  | Positive | Negative | Total |
| Serum | Positive | 2 | 0 | 2 |
|  | Negative | 0 | 30 | 30 |
|  | Total | 2 | 30 | 32 |

**Supplemental Table 5: Head Computed Tomography Results by *Cryptococcal* Antigen Test (CrAg)**

|  | | Cryptococcus | | | | | |
| --- | --- | --- | --- | --- | --- | --- | --- |
|  |  | Positive N=9 | Negative N73 |  | Poisson regression with robust variance | | |
|  |  | N(%) / Median(IQR) | N(%) / Median(IQR) | p-value^a^ | PR | 95% CI | p-value |
| **Computed Tomography Findings** | |  |  |  |  |  |  |
| Lesions (% Yes) | | 2/9 (22%) | 47/73 (64%) | **0.02** | 0.19 | 0.04-0.88 | **0.03** |
| Number of Lesions | |  |  |  |  |  |  |
|  | None | 7 (21%) | 26 (79%) | 0.10 | Ref. | - | - |
|  | 1 | 1 (5%) | 20 (95%) |  | 0.22 | 0.03-1.72 | 0.15 |
|  | 2-5 | 0 | 20 (100%) |  | - | - | **-** |
|  | 6-10 | 1 (17%) | 5 (83%) |  | 0.79 | 0.12-5.35 | 0.81 |
|  | 11-20 | 0 | 2 (100%9 |  | - | - | **-** |
| Number Hyperdense Lesions | | | | | | | |
|  | None | 8 (13%) | 54 (87%) | 0.87 | Ref. | - | - |
|  | 1 | 1 (9%) | 10 (91%) |  | 0.71 | 0.10-5.15 | 0.73 |
|  | 2-5 | 0 | 7 (100%) |  | - | - | **-** |
|  | 6-10 | 0 | 1 (100%) |  | - | - | **-** |
|  | 11-20 | 0 | 1 (100%) |  | - | - | **-** |
| Number Hypodense Lesions | | | | | | | |
|  | None | 7 (16%) | 36 (84%) | 0.29 | Ref. | - | - |
|  | 1 | 1 (6%) | 15 (94%) |  | 0.38 | 0.05-2.92 | 0.36 |
|  | 2-5 | 0 | 17 (100%) |  | - | - | **-** |
|  | 6-10 | 1 (20%) | 4 (80%) |  | 1.23 | 0.19-8.14 | 0.83 |
|  | 11-20 | 0 | 1 (100%) |  | - | - | **-** |
| Number Affected Hemispheres | | | | | | | |
|  | None | 7 (21%) | 26 (79%) | 0.05 | Ref. | - | - |
|  | One | 1 (3%) | 28 (97%) |  | 0.16 | 0.02-1.26 | 0.08 |
|  | Two | 1 (5%) | 19 (95%) |  | 0.24 | 0.03-1.78 | 0.16 |
| Edema | | | | | | | |
|  | None | 7 (17%) | 33 (83%) | 0.44 | Ref. | - | - |
|  | Minor | 1 (5%) | 21 (95%9 |  | 0.26 | 0.03-2.00 | 0.20 |
|  | Moderate | 1 (7%) | 14 (93%) |  | 0.38 | 0.05-2.88 | 0.35 |
|  | Severe | 0 | 5 (100%) |  | - | - | - |
| Mass Effect | | | | | | | |
|  | None | 8 (13%) | 52 (87%) | 0.86 | Ref. | - | - |
|  | Minor | 1 (8%) | 11 (92%) |  | 0.63 | 0.09-4.60 | 0.65 |
|  | Moderate | 0 | 9 (100%) |  | - | - | - |
|  | Severe | 0 | 1 (100%) |  | - | - | - |
| Hydrocephalus (%Yes) | | 0/9 (0%) | 10/73 (14%) | 0.59 | - | - | - |
| White Matter Changes (%Yes) | | 2/9 (22%) | 22/73 (30%) | 0.62 | 0.69 | 0.15-3.12 | 0.63 |
| Type of White Matter Changes | | | | | | | |
|  | None | 7 (12%) | 51 (88%) | 1.00 | Ref. | - | - |
|  | Minor | 1 (8%) | 11 (92%) |  | 0.69 | 0.09-5.17 | 0.72 |
|  | Moderate | 1 (10%) | 9 (90%) |  | 0.83 | 0.11-6.11 | 0.85 |
|  | Generalized | 0 | 2 (100%) |  | - | - | **-** |
| a: p-values were calculated with chi-square tests or fisher exact test for categorical variables and Mann Whitney U test for numerical variables | | | | | | | |
|  |  |  |  |  |  |  |  |
|  |  |  |  |  |  |  |  |
|  |  |  |  |  |  |  |  |

**Supplemental Table 6: Head Computed Tomography Results by Survival**

|  | | Survival | | | | | | |  |
| --- | --- | --- | --- | --- | --- | --- | --- | --- | --- |
|  |  | Alive N=37 | Dead N=41 | |  | Poisson regression with robust variance | | |  |
|  |  | N(%) / Median(IQR) | N(%) / Median(IQR) | | p-value^a^ | PR | 95% CI | p-value |  |
| **Computed Tomography Findings** | | | | | | | | | |
| Lesions (% Yes) | | 18/37 (49%) | 30/41 (73%) | | **0.03** | 1.71 | 1.01-2.87 | **0.05** |  |
| Number of Lesions | | | | | | | | | |
|  | None | 19 (63%) | 11 (37%) | 0.09 | | Ref. | - | - |  |
|  | 1 | 10 (50%) | 10 (50%) |  |  | 1.36 | 0.71-2.60 | 0.35 |  |
|  | 2-5 | 6 (32%) | 13 (68%) |  |  | 1.87 | 1.06-3.28 | **0.003** |  |
|  | 6-10 | 1 (14%) | 6 (86%) |  |  | 2.34 | 1.33-4.10 | **0.003** |  |
|  | 11-20 | 1 (50%) | 1 (50%) |  |  | 1.36 | 0.31-5.95 | 0.68 |  |
| Number Hyperdense Lesions | | | | | | | | | |
|  | None | 29 (48%) | 31 (52%) | | 0.68 | Ref. | - | - |  |
|  | 1 | 3 (33%) | 6 (67%) | |  | 1.29 | 0.76-2.18 | 0.34 |  |
|  | 2-5 | 4 (57%) | 3 (43%) | |  | 0.83 | 0.34-2.03 | 0.68 |  |
|  | 6-10 | 0 | 1 (100%) | |  | - | - | **-** |  |
|  | 11-20 | 1 (100%) | 0 | |  | - | - | **-** |  |
| Number Hypodense Lesions | | | | | | | | | |
|  | None | 24 (63%) | 14 (37%) | | **0.03** | Ref. | - | - |  |
|  | 1 | 7 (47%) | 8 (53%) | |  | 1.45 | 0.77-2.73 | 0.25 |  |
|  | 2-5 | 5 (28%) | 13 (72%) | |  | 1.96 | 1.18-3.26 | **0.01** |  |
|  | 6-10 | 1 (17%) | 5 (83%) | |  | 2.26 | 1.30-3.93 | **0.004** |  |
|  | 11-20 | 0 | 1 (100%) | |  | - | - | **-** |  |
| Number affected Hemispheres | | | | | | | | | |
|  | None | 20 (67%) | 10 (33%) | | **0.02** | Ref. | - | - |  |
|  | One | 11 (42%) | 15 (58%) | |  | 1.73 | 0.94-3.18 | 0.08 |  |
|  | Two | 6 (27%) | 16 (73%) | |  | 2.18 | 1.23-3.86 | **0.01** |  |
| Edema | | | | | | | | | |
|  | None | 20 (54%) | 17 (46%) | | 0.52 | Ref. | - | - |  |
|  | Minor | 8 (36%) | 14 (64%) | |  | 1.39 | 0.86-2.23 | 0.18 |  |
|  | Moderate | 7 (44%) | 9 (56%) | |  | 1.22 | 0.7-2.14 | 0.48 |  |
|  | Severe | 2 (67%) | 1 (33%) | |  | 0.73 | 0.14-3.77 | 0.70 |  |
| Mass Effect | | | | | | | | | |
|  | None | 28 (50%) | 28 (50%) | | 0.46 | Ref. | - | - |  |
|  | Minor | 4 (31%) | 9 (69%) | |  | 1.39 | 0.88-2.17 | 0.16 |  |
|  | Moderate | 4 (50%) | 4 (50%) | |  | 1.00 | 0.47-2.11 | 1.00 |  |
|  | Severe | 1 (100%) | 0 | |  | - | - | **-** |  |
| Hydrocephalus (%Yes) | | 5/37 (14%) | 3/41 (7%) | | 0.37 | 0.69 | 0.27-1.74 | 0.43 |  |
| White Matter Changes (%Yes) | | 12/37 (32%) | 10/41 (24%) | | 0.43 | 0.82 | 0.49-1.38 | 0.46 |  |
| Type of White Matter Changes | | | | | | | | | |
|  | None | 25 (45%) | 31 (55%) | | 0.44 | Ref. | - | - |  |
|  | Minor | 7 (64%) | 4 (36%) | |  | 0.66 | 0.29-1.49 | 0.32 |  |
|  | Moderate | 4 (40%) | 6 (60%) | |  | 1.08 | 0.62-1.9 | 0.78 |  |
|  | Generalized | 1 (100%) | 0 | |  | - | - | **-** |  |
| a: p-values were calculated with chi-square tests or fisher exact test for categorical variables and Mann Whitney U test for numerical variables | | | | | | | | | |
